# Comparative Analysis of the Transcriptomes of Persisting and Abscised Fruitlets: Insights into Plant Hormone and Carbohydrate Metabolism Regulated Self-Thinning of Pecan Fruitlets during the Early Stage

**DOI:** 10.3390/cimb44010013

**Published:** 2021-12-30

**Authors:** Jiyu Zhang, Tao Wang, Fan Zhang, Yongzhi Liu, Gang Wang

**Affiliations:** Institute of Botany, Jiangsu Province and Chinese Academy of Sciences, Nanjing 210014, China; maxzhangjy@163.com (J.Z.); immmorer@163.com (T.W.); mumizhongfeng@126.com (F.Z.); liuyz1965@163.com (Y.L.)

**Keywords:** pecan, abscised fruitlet, plant hormone, carbohydrate metabolism

## Abstract

Pecan is one of the most popular nut species in the world. The fruit drop rate of the pecan ‘Pawnee’ is more than 57%, with four fruit drop stages, which is very serious. In this study, we conducted transcriptomic profiling of persisting and abscised fruitlets in early fruit development by RNA-seq. A total of 11,976 differentially expressed genes (DEGs) were identified, 3012 upregulated and 8964 downregulated, in a comparison of abscised vs. persisting fruitlets at 35 days after anthesis (DAA). Our transcriptomic data suggest that gene subsets encoding elements involving the biosynthesis, metabolism, perception, signal transduction, and crosstalk of the plant hormones abscisic acid (ABA), auxin, cytokinin, ethylene, and gibberellin (GA) and plant growth regulators jasmonates, salicylic acid, and brassinosteroids were differentially expressed. In addition, the majority of transcriptionally activated genes involved in hormone signaling (except for ethylene and salicylic acid signaling) were downregulated in abscised fruitlets. The differential expression of transcripts coding for enzymes involved in sucrose, glucose, trehalose, starch, galactose, and galactinol metabolism shows that sucrose, galactinol, and glucose synthesis and starch content were reduced as starch biosynthesis was blocked, and retrogradation and degradation intensified. These results suggest that the abscised pecan fruitlets stopped growing and developing for some time before dropping, further indicating that their sugar supply was reduced or stopped. The transcriptome characterization described in this paper contributes to unravelling the molecular mechanisms and pathways involved in the physiological abscission of pecan fruits.

## 1. Introduction

Vegetative and reproductive organs that are senescent, infected, or damaged are shed from the main plant body, which is called abscission. Abscission is a specific sequence of highly complex regulated events [1]. The regulatory network activated by the abscising organ leads to the activation of abscission zones. To simplify the explanation, the abscission process includes four phases. The first step is the development of an abscission zone. The next step is the activation of abscission signaling. Then, enzymatic hydrolysis takes place in the middle lamella of the abscission zone (AZ), and AZ cells begin to enlarge. The last step is the further differentiation 1and sealing of the abscission scar [2].

Fruitlet abscission, especially for the so-called physiological drop or June drop, is very common in fruit tree development in order to control fruit load according to the nutritional status, allowing the plant to make efficient use of resources [3]. The molecular mechanisms that control and lead to early physiological fruitlet abscission are studied in model apple [3,4,5,6] and tomato [7,8] plants. The progress of physiological drop differs from senescence-driven abscission at or after ripening. Dropping of young fruits during early phases of development can be understood as a developmental response by which the plant selectively abscises fruitlets that represent weaker sinks to adapt to nutritional shortages [5]. The molecular mechanisms regulating early fruit development and the plasticity of fruit development in response to endogenous and environmental changes can be understood by studying fruit physiological drop in this scenario.

Fruit set is the process of ovary tissues undergoing transformation into fruit. It is well known that auxins and gibberellins (GAs) play a critical role in the inductive phase of fruit set. A few studies have shown that auxins trigger cell division, and their interplay with GAs sustain cell expansion [9]. However, the transformation of the ovary into fruit of tomato and *Arabidopsis* is prevented by a negative control exerted by auxin/indole acetic acid (AUX/IAA) and auxin response factor (ARF) proteins. This negative regulation can be removed through pollination/fertilization or treatment with auxins, leading to cell proliferation and fruit set. Ethylene and abscisic acid (ABA) biosynthesis and action appear to be significantly downregulated, and concomitantly those of auxin and GA biosynthesis and action are activated as soon as fruit set is triggered [10,11]. This suggests that plant hormones play an important role in fruit development. A few reports have shown that plant hormones auxin, ethylene, and ABA seem to play major roles, and GAs, cytokinins, and jasmonates have also been reported to be involved [3,12,13].

Sugar metabolism also plays a major role in the response to the progress of fruitlet abscission. The contents of sorbitol, glucose, fructose, and sucrose were shown to be lower in abscising fruitlets than in persisting fruitlets [6]. Transcriptomic analysis showed that genes coding trehalose-6-phosphate synthase, sorbitol transporter, UDP-glucosyltransferases, and UDP-Glc-4-epimerase were upregulated in abscising apple fruitlet [5]. The class of enzyme genes is also controlled by sugar starvation and involved in resource mobilization in other species [14,15,16].

Pecans (*Carya illinoinensis* (Wangenh.) K. Koch), which are native to North America and belong to the Juglandaceae family, are the most economically valuable nut trees in the world [17,18]. Pecan trees exhibit a strong tendency to produce a heavy crop one year and a light crop the following year. It is sometimes difficult to compile cultivation recommendations to minimize alternate bearing. Despite years of research, the physiology behind alternate bearing is only partially understood, and this phenomenon remains the principal challenge faced by the pecan industry year after year. It is very important to make clear the process of fruit development and the mechanism of natural fruit abscission in pecan in order to control a reasonable load. Thus, transcriptomes from persisting and abscised fruitlets were sequenced and differentially expressed genes between them were identified in order to fully understand the differences in gene expression and explore the cause of fruitlet abscission in pecan. The contents of plant hormones of persisting and abscised fruitlets were measured in this study. This study can serve to broaden our understanding of the mechanism of fruit development and natural fruit abscission.

## 2. Experimental Section

### 2.1. Plant Materials, Fruit Development, and Drop Dynamics Analysis

Experiments were carried out over 2 years on 10-year-old pecan trees (Pawnee) with spacing of 6 m × 8 m, grown with standard horticultural practices at the experimental farm of the Institute of Botany, Jiangsu Province, and Chinese Academy of Sciences (32°18′ N; 118°52′ E). Fruit drop was monitored from 7 DAA until fruit maturity at weekly intervals using 50 pre-marked fruit clusters from each of 5 homogeneous trees. Further, 10 fruits were sampled from each of 5 additional homogeneous trees from 7 DAA until fruit maturity at weekly intervals to study fruit development. Finally, 10 naturally abscised and persisting fruitlets were harvested at 36 and 48 DAA from each of 3 homogeneous trees for the study reported in this paper.

### 2.2. RNA Isolation and cDNA Library Preparation and Sequencing

Naturally abscised and persisting fruitlets harvested at 36 DAA were used for transcriptome analysis. Total RNA was extracted from the fruitlets using a Trizol reagent kit (Invitrogen, Carlsbad, CA, USA) according to the manufacturer’s protocol. The individual RNA samples were analyzed spectrophotometrically for protein contamination, and the samples with A260/A280 ratio values ranging from 1.9 to 2.0 were chosen. After total RNA was extracted, mRNA was enriched by oligo (dT) beads. The enriched mRNA was then fragmented into short fragments and reverse transcribed into cDNA by using the NEBNext Ultra RNA Library Prep Kit for Illumina (NEB#E7530, New England Biolabs, Ipswich, MA, USA). The purified double-stranded cDNA fragments were end repaired, poly(A) was added, and they were ligated to Illumina sequencing adapters. The ligation reaction was purified with AMPure XP Bead (1.0×). Ligated fragments were subjected to size selection by agarose gel electrophoresis and amplified PCR. The resulting cDNA library was sequenced using an Illumina HiSeq 2500 by Genedenovo Biotechnology Co., Ltd. (Guangzhou, China). The raw sequencing data generated from this study have been deposited in NCBI SRA (http://www.ncbi.nlm.nih.gov/sra, last accessed on 1 December 2021) under accession number PRJNA784729.

### 2.3. Analysis of Differential Gene Expression

Clean reads were obtained by filtering the raw reads, including adapters or low-quality bases, by fastp (v.18) [19]. The clean reads were mapped to a ribosome RNA database using the Bowtie2 (v.2.28) short read alignment tool [20] to identify and remove residual rRNA reads. The remaining clean reads were mapped to the pecan genome (Cil.genome.fa, ftp://parrot.genomics.cn/gigadb/pub/10.5524/100001_101000/100571/) using HISAT2.2.4 [21] with the parameter “–rna-strandness RF” and default settings for the other parameters. The unmapped reads were assembled by using StringTie v.1.3.1 [22], and the assembled sequences were regarded as novel genes. HTSeq (v.0.6.0) [23] was used to calculate the raw read counts for each gene. The gene expression was calculated and normalized to reads per kilobase per million mapped reads (RPKM) [24]. Analysis of gene expression differences between two groups was performed by DESeq2 software (European Molecular Biology Laboratory, Heidelberg, Germany) [25]. Transcripts with a false discovery rate (FDR) < 0.05 and absolute fold change > 2 were defined as differentially expressed genes (DEGs). Gene Ontology (GO) enrichment (*p*-value < 0.05) was studied by running all DEGs through the GO database (http://www.geneontology.org/) to further classify the genes or their products into terms (molecular function, biological process, and cellular component) to understand their biological functions. Pathway projects were performed according to the KEGG pathway database for pathway enrichment analysis of DEGs.

### 2.4. Plant Hormone Content Analysis

Approximately 200 mg of normal persisting fruitlets and abscised fruitlets (just shed from the main plant body) were used to measure the levels of plant hormones (ABA, IBA, GA, and TZR). Plant hormones were detected by high-performance liquid chromatography coupled with electrospray ionization tandem mass spectrometry (HPLC-ESI-MS/MS) on an Agilent 1290 HPLC (Agilent Technologies Inc., Santa Clara, CA, USA) and a Sciex QTRAP 6500 mass spectrometer (AB Sciex LLC, Framingham, MA, USA). The experiment was performed by Nanjing Innovation Biotechnology Co., Ltd. (Nanjing, China). Three biological replicates were analyzed for this experiment, each one containing 30 fruitlets. Statistically significant differences of plant hormones were calculated with GraphPad Prism 7.0 software (San Diego, CA, USA).

## 3. Results

### 3.1. Analysis of Pecan Fruit Drop Dynamics

**Figure 1:**
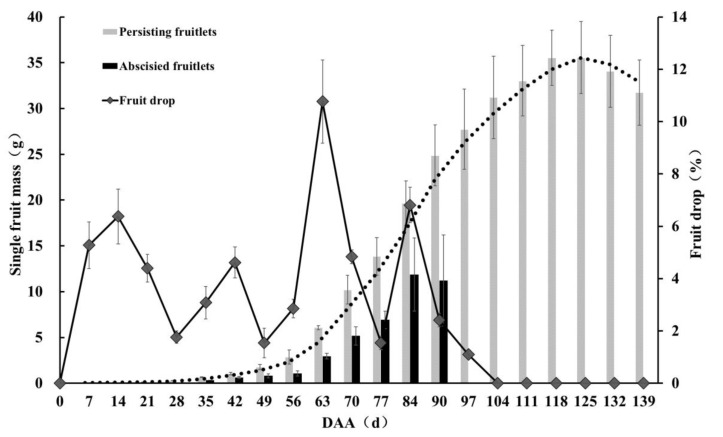
Analysis of pecan ‘Pawnee’ fruit development and drop dynamics. Black dot line indicates the fruit development dynamic trend. Mean values (±SD) of three biological replicates are shown. DAA, days after anthesis.

Pecan ‘Pawnee’ fruit drop and fruit development dynamics were investigated in this study (Figure 1). The results show that fruit growth exhibited the characteristic of a single sigmoid growth curve, presenting only one rapid growth period. The fruit drop rate was more than 57%, with four fruit drop stages, occurring at 0–28 days after anthesis (DAA), 28–49 DAA, 49–77 DAA, and 77–104 DAA. The fruit drop ratio was the largest at the third fruiting stage, accompanied by a rapid fruit growth period. The single fruit mass of persisting fruitlets was about twice that of abscised fruitlets (just shed from the main plant body) (Figure 1 and S1), indicating that the abscised fruitlets had stopped growing and developing for some time before dropping.

### 3.2. RNA Sequencing of Transcriptomes of Persisting and Abscised Fruitlets and Mapping of RNA Sequences to Reference Genome

To test the differentially expressed genes of persisting fruitlets (P1) and abscised fruitlets (A1) in the early stage of fruit development (35 DAA), two groups with six samples were analyzed by RNA-seq, and a total of 35.66 GB of clean data was generated by strict quality control and processing (Supplementary Table S1). Details on data and data quality before and after filtering are shown in Table 1 and Supplementary Table S1. In all, 112, 434, and 202 reads and 135, 904, and 652 reads were generated for abscised and persisting fruitlets, respectively, after filtering out duplicate sequences and ambiguous and low-quality reads (Supplementary Table S1). The numbers of clean bases and reads of persisting fruitlets were more than those of abscised fruitlets (Table 1 and Supplementary Table S1). The average GC percentage was 45.41%, with a QC30 base percentage above 93.05%. HQ clean reads were mapped to the pecan reference genome (Cil.genome.fa). Approximately 38.94 million clean reads (94.11% of the total) were mapped, and the number of mapped reads (42.57 × 10^6^) of persisting fruitlets was more than that of abscised fruitlets (35.32 × 10^6^). This indicates that the number of transcripts of persisting fruitlets was more than that of abscised fruitlets. A total of 28,624 genes were obtained from 6 transcriptome libraries, including 26,537 reference genes and 2087 novel genes (Supplementary Table S2). The numbers of reference genes, novel genes, and total genes of persisting fruitlet transcripts were more than those of abscised fruitlet transcripts (Table 1 and Supplementary Table S1).

### 3.3. Differentially Expressed Gene Analysis

In our study, we calculated the correlation coefficient between the samples to test sample repeatability. The correlation coefficient in the repeat group was greater than 0.808 (Supplementary Figure S2), indicating consistency among the three biological replicates. Thus, the RNA-seq results were confirmed to be highly reliable for further analysis. A total of 11,976 DEGs (about 41.84% of total sequenced genes) were identified in the comparison of abscised fruitlets vs. persisting fruitlets at 35 DAA; among them, 3012 DEGs (25.15%) were upregulated and 8964 DEGs (74.85%) were downregulated (Supplementary Tables S3 and S4). There were about three times as many downregulated genes as upregulated genes, indicating that the vital signs of abscised fruitlets had diminished.

**Figure 2:**
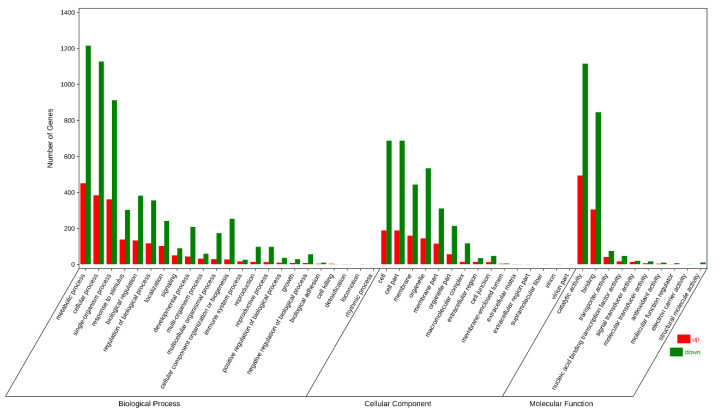
GO functional classification annotation of differentially expressed genes of pecan in comparison of abscised fruitlets vs. persisting fruitlets at 35 DAA.

**Figure 3:**
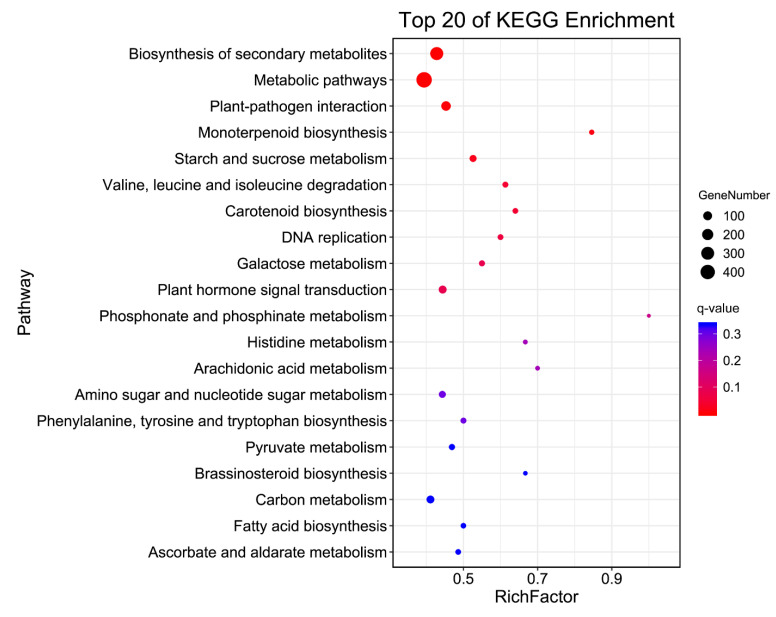
Top KEGG pathways mapping enriched differential progress.

Gene Ontology (GO) functional classification included three GO trees (cellular components, molecular functions, and biological processes) and 48 functional groups (Figure 2 and Supplementary Table S5). In the category of biological processes, the largest groups were metabolic, cellular, and single-organism processes. For the cellular components, DEGs with cell, cell part, membrane, and organelle formed the major groups. Catalytic activity and binding were the dominant groups in the molecular function category. The top five enrichment categories in GO were catalytic activity, cellular process, single-organism process, primary metabolic process, and single-organism cellular process (Supplementary Table S6). In order to understand their biological function, all of the DEGs were also mapped to terms in the Kyoto Encyclopedia of Genes and Genomes (KEGG) database. Finally, 1050 DEGs were matched and assigned to 129 KEGG pathways (Supplementary Table S7). The first five biological pathways, involving biosynthesis of secondary metabolites, metabolic pathways, plant–pathogen interaction, monoterpenoid biosynthesis, and starch and sucrose metabolism, were significantly enriched in persisting fruitlets (P1) and abscised fruitlets (A1) (Figure 3 and Table 2).

### 3.4. Identification of Differentially Expressed Proteins Involved in Plant Hormone Signal Transduction

Gene subsets encoding elements involved in hormone biosynthesis, metabolism, perception, signal transduction, and crosstalk were found to be differentially expressed in the comparison of abscised fruitlets vs. persisting fruitlets at 35 DAA using de novo transcriptome sequencing (Table 3 and Supplementary Table S8). The statistical analyses were performed for plant hormones ABA, auxin, cytokinin, ethylene, and GA and plant growth regulators jasmonates, salicylic acid, and brassinosteroids. All 3 identified DEGs of the ABA metabolic process, 7 DEGs in response to ABA, and 8 out of 9 DEGs involved in ABA signal transduction were downregulated in abscised fruitlets vs. persisting fruitlets. All 6 identified DEGs involved in the auxin metabolic process, 7 out of 10 DEGs involved in auxin transport, and 15 out of 20 DEGs involved in auxin signal transduction were downregulated. Half of the identified DEGs involved in the gibberellin metabolic process and 10 out of 12 DEGs involved in cytokinin signal transduction were downregulated. Two out of 3 identified DEGs involved in the gibberellin metabolic process, all 4 DEGs involved in the gibberellin-mediated signaling pathway, and 2 out of 3 DEGs involved in gibberellin signal transduction were downregulated. All 3 identified DEGs involved in ethylene signal transduction were upregulated. These results show that almost all identified DEGs involved in ABA, auxin, cytokinin, and gibberellin were downregulated, and all identified DEGs involved in ethylene were upregulated. Eleven out of 20 identified DEGs involved in the SA metabolic process and 5 out of 6 DEGs involved in SA signal transduction were upregulated, but, all 9 identified DEGs involved in the JA metabolic process and 5 out of 7 DEGs involved in JA signal transduction were downregulated, indicating that SA biosynthesis and the signal transduction pathway were promoted, but were suppressed in abscised fruitlets of pecan.

### 3.5. Key Identified Differentially Expressed Proteins Involved in Starch and Sucrose Metabolism and Galactose Metabolism

Our analysis reveals that the transcriptional activity of genes involved in starch and sucrose metabolism and galactose metabolism were significantly regulated in the abscised fruitlets of pecan (Table 4 and Supplementary Table S4). The differential expression of transcripts coding for enzymes involved in sucrose, glucose, trehalose, starch, galactose, and galactinol metabolism was analyzed. For sucrose metabolism, the expression of two sucrose phosphate synthase (SPS) genes (EC: 2.4.1.14) were downregulated and *CWINV1* (invertase, EC: 3.2.1.26) expression was upregulated in abscised fruitlets of pecan. All 4-glucan endo-1,3-beta-glucosidase genes (EC: 3.2.1.39), 6 out of 10 detected genes coding *β*-glucosidases (EC: 3.2.1.21), and an endoglucanase gene (EC: 3.2.1.4) for glucose metabolism were downregulated in abscised fruitlets compared with persisting fruitlets. For starch metabolism, two granule-bound starch synthase 1 (GBSS1, EC: 2.4.1.242), one GBSS2, and two 1,4-a-glucan branching enzyme (GBE, EC: 2.4.1.18) genes were downregulated in abscised fruitlets; however, two starch degradation alpha-amylase (EC: 3.2.1.1) genes were upregulated. For trehalose metabolism, the expression of trehalose 6-phosphate synthase (TPS, EC: 2.4.1.25) and trehalose-6-phosphate phosphatase (TPP, EC: 3.1.3.12) was higher in abscised fruitlets than in persisting fruitlets. The expression of alpha-galactosidase gene (EC: 3.2.1.22) was downregulated, but the expression of aldose 1-epimerase gene (EC: 5.1.3.3) was enhanced for galactose synthesis metabolism. Three out of 4 inositol 3-alpha-galactosyltransferases (GolS, EC: 4.1.123) were downregulated in abscised pecan fruitlets.

### 3.6. Plant Hormone Content Analysis

**Figure 4:**
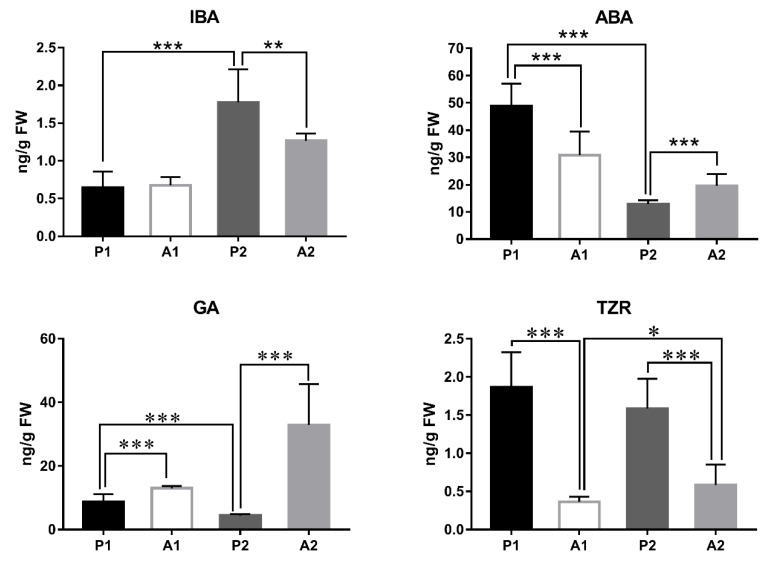
Analysis of plant hormone content in abscised and persisting fruitlets at 35 and 48 DAA. P1, persisting fruitlets at 35 DAA; P2, persisting fruitlets at 48 DAA; A1, abscised fruitlets at 35 DAA; A2, abscised fruitlets at 48 DAA. Statistically significant differences were calculated with GraphPad Prism 7.00. Data represent average ± SD of three biological repeats with three measurements. * Significant differences at *p* < 0.05; ** significant differences at *p* < 0.01; *** significant differences at *p* < 0.001.

The contents of plant hormones 3-indolebutyric acid (IBA), ABA, GA, and Trans-zeatin riboside (TZR) were measured in abscised and persisting fruitlets at 35 and 48 DAA to further understand changes in plant hormone content after fruit shedding in pecan (Figure 4). As the fruit developed, the IBA content increased significantly, but ABA and GA content decreased significantly, indicating that increased IBA content and decreased ABA and GA might promote fruit development in pecan. Compared with persisting fruitlets, the IBA content was not changed in abscised fruitlets at 35 DAA but decreased significantly at 48 DAA. The ABA content was significantly lower in abscised fruitlets than in persisting fruitlets at 36 DAA, but the opposite was the case at 48 DAA. The GA content was significantly higher in abscised fruitlets than in persisting fruitlets, and the TZR content was significantly lower in abscised fruitlets than in persisting fruitlets at 36 and 48 DAA.

### 3.7. Response of Transcription Factors in the Comparison of A1 vs. P1

Differential expression of transcription factor genes was analyzed to identify transcription factors involved in abscised fruitlets in pecan (Table 4 and Supplementary Table S9). We identified 40 MYB transcription factors with significantly differential expression, 10 upregulated and 30 downregulated, suggesting that these factors might be involved in abscising in pecan. Among the NAM, ATAF1–2, and cup-shaped cotyledon 2 (NAC) transcription factor family, 13 members were upregulated and 11 members were downregulated in abscised compared with persisting fruitlets. WRKY, basic helix-loop-helix protein (bHLH), basic region/ leucine zipper motif (bZIP), C2C2, C2, ethylene response factor (ERF), zinc finger, and B3 transcription factor families were up- or downregulated in abscised fruitlets vs. persisting fruitlets, suggesting that these families may also play key roles in the transcriptional regulation of genes in abscission in the early stage of fruit development in pecan.

## 4. Discussion

### 4.1. Increased IBA Content and Decreased ABA and GA Might Promote Fruit Development in Pecan

Previous reports showed that the ethylene and ABA molecular biosynthesis and action mechanisms appear to be significantly downregulated as soon as fruit set is triggered, and concomitantly, auxin and GA molecular biosynthesis and action mechanisms are activated [10,11], indicating that fruit development appears to rely on the removal of a negative feedback regulation established by a negative control exerted mainly by ABA- and ethylene-dependent pathways of ovary growth [11]. Auxins may trigger cell division, and their interaction with GAs may be essential for sustaining cell expansion [9]. In this study, the IBA content was increased significantly, but the ABA and GA contents were decreased significantly as the fruit developed at the early stage (Figure 4), indicating that increased IBA and decreased ABA and GA might promote fruit development in pecan. Thus, further studies are needed on the molecular role of GA in the development of pecan.

### 4.2. Plant Hormones Seem to Play a Key Role during the Abscission Progress in Pecan

The IBA content of abscised fruitlets did not change abruptly at 35 DAA compared with persisting fruitlets, but decreased significantly at 48 DAA; however, the ABA content decreased significantly at 35 DAA but increased abruptly at 48 DAA, and the GA content was significantly higher in abscised fruitlets than in persisting fruitlets. These results show that the changes in plant hormones in abscised and persisting fruitlets have different trends at different stages of development in pecan. At 42 DAA, increased ABA and GA and decreased IBA and TZR might promote fruitlet abscission in pecan, but downregulation of GA signaling specifically in fruits induced to abscise in apple [5]. At 35 DAA, decreased ABA and no change in IAA were observed by comparing their contents in abscised and persisting fruitlets. Further studies are needed to study the initiation of abscission in pecan regarding plant hormone signaling, especially GAs, at the early stage.

Our transcriptomic data suggest that the majority of transcriptionally activated genes involved in hormone signaling were downregulated in abscised fruitlets, except for ethylene and salicylic acid signaling (Table 3 and Supplementary Table S8), indicating that hormones may play an important role during the progress of abscission in pecan. Botton et al. reported that ABA and ethylene signaling were strongly upregulated concurrently, with downregulation of GA signaling specifically in fruits induced to abscise in apple [5]. The essential role of ABA in abscission has been broadly studied in different species [4]. Exogenous ABA treatment can induce fruitlet abscission in the apple L3 class, and ABA may be involved in upstream induction of abscission [4]. Increased levels of ABA observed in BA-treated apple L3 fruitlets that were abscising, concurrently with upregulation of the *ABA*-responsive *9*-*cis*-*epoxycarotenoid dioxygenase 1* (*MdNCED1*) gene [26], suggest activation of the indirect biosynthetic pathway of ABA [4]. In our study, two *NCED* encoding genes were downregulated in abscised pecan fruitlets (Supplementary Table S8), consistent with the decreased ABA content (Figure 4). In addition, 8 ABA signal transduction genes were downregulated in abscised pecan fruitlets. These results show that ABA signaling is strongly downregulated in abscised pecan fruitlets, but on the contrary, it is strongly upregulated in apple fruits induced to abscise [5].

Ethylene signaling was significantly enhanced in abscised pecan fruitlets, as shown by high expression of some ethylene signaling genes (Table 3). *Mitogen*-*activated protein kinase* (*MAPK*) genes, which were found to be upregulated in abscised fruitlets (Supplementary Table S8), are key elements of the ethylene signal transduction pathway, probably involved in ethylene–ABA crosstalk [27]. The related proteins (*14*-*3*-*3* genes), which were also found to be upregulated in abscised fruitlets (Supplementary Table S8), may trigger ABA–ethylene crosstalk and the response to sugar starvation [28]. The *TPS* gene, found to be overexpressed in abscised fruitlets, may also regulate ABA signaling, as found in *Arabidopsis* [29] and apple [5]. ABA and ethylene signaling are strongly upregulated concurrently with downregulation of GA signaling specifically in apple fruits induced to abscise [5]. Our transcriptomic data show that ethylene signaling is strongly upregulated concurrently with downregulation of GA and ABA signaling in abscised pecan fruitlets.

Auxin participates in plant organ abscission. Transcript analysis revealed that auxin may regulate the expression of early auxin-responsive gene families including *AUX*/*IAA*, *gretchen Hagen3* (*GH3*), and *small auxin up RNA* (*SAUR*). Auxin regulates the expression of various ARFs during early abscission, whereas ethylene has the opposite effect on most of these genes in tomato [30]. SlARFs have overlapping functions in the abscission process. Meir et al. reported that tomato flower abscission was associated with the expression level of *AUX*/*IAA* genes [31]. *GH3* genes are also involved in fruitlet or flower abscission. *LcAUX/IAA1* and *LcSAUR1* may play more important roles in abscission than *LcGH3.1* in litchi, because *LcAUX/IAA1* and *LcSAUR1* were mostly expressed in AZ [32]. Tomato *GH3* increased slightly after 8 h and maintained a low expression level during abscission, implying that it may be an effective negative regulator in IAA-induced abscission delay [33]. *SAUR*, an auxin-responsive gene, may serve as a marker of IAA level throughout the abscission process [33]. Two *SAUR*-like genes were found to be involved in shading-induced abscission by transcriptomic analysis in apple [34]. Girdling plus defoliation treatment could induce *LcSAUR1* expression in AZ and fruitlet, and significantly induce litchi fruitlet abscission [32]. SAUR36 has been reported to be involved in leaf senescence in *Arabidopsis* [35]. Overexpression of *OsSAUR39* in rice (*Oryza sativa*) results in phenotypes that include reductions in lateral root development, yield, and shoot and root length, suggesting that *OsSAUR39* acts as a negative regulator of auxin synthesis and transport [36]. Fourteen *CitSAUR* genes showed obvious changes during citrus fruitlet abscission, and *CitSAUR06*, *CitSAUR08*, *CitSAUR44*, *CitSAUR61*, and *CitSAUR64* were more relevant because their expression patterns under IAA treatment exhibited an opposite trend to that during fruitlet drop [37]. In our study, all 8 *AUX*/*IAA*, 3 *ARF*, and 2 out of 3 *GH3* genes detected were downregulated, but 3 out of 4 *SAUR* genes were upregulated in abscised pecan fruitlets. These results show that the roles of *GH3*, *ARF*, *AUX*/*INN*, and *SAUR* in fruitlet abscission may vary considerably among plant species.

Beyond the GA, ABA, auxin, and ethylene signaling discussed above, cytokinin, jasmonate, salicylic acid, and brassinosteroid signaling are also involved in abscised pecan fruitlets. An association of response variables with expression data of genes regarding cytokinin, jasmonates, salicylic acid, and brassinosteroids was detected in abscising apple fruitlets [5]. Especially related to salicylic acid, many genes related to metabolism were highly expressed in abscised fruitlets. A *TGA* gene and 4 *PR*-*1* genes were upregulated in abscised fruitlets, indicating that systemic acquired resistance (SAR) may be triggered in abscised pecan fruitlets.

### 4.3. Reduced Sugar Supply in Abscised Fruitlets Is One Reason for Fruitlet Abscission in Pecan

Sugar signaling is known to play a role in senescence regulation in a complex network. Thus, the senescence process is triggered in fruitlets once the sugar supply is decreased [38]. In apple, the sorbitol concentration of central fruitlets is higher than that of L1 pedicels, indicating that the former profit from a better supply of sugars. The increased supply of sugar to the central fruit would allow it to develop at a faster rate, thus further increasing its sink strength [6]. In our study, the single fruit mass of abscised fruitlets (just shed from the main plant body) was about half that of persisting fruitlets (Figure 1 and Supplementary Figure S1), indicating that abscised fruitlets had stopped growing and developing for some time before dropping, thus further indicating that the sugar supply is reduced or stopped in abscising pecan fruitlet.

The transcriptome analysis showed that genes coded for carbohydrate metabolism, including starch and sucrose, galactose, pyruvate, and amino sugar and nucleotide sugar metabolism, are significantly regulated in abscised pecan fruitlets (Table 5). Decreased expression of transcripts coding for enzymes involved in sucrose, glucose, and starch metabolism may be expected in slow-growing organs, considering that fruitlets act as a major carbon sink. SPS catalyzing fructose-6-phophate and UDP-glucose to synthesis sucrose-6-phosphate in plants is a key regulatory step in the control of sucrose synthesis [39]. Overexpression of *SPS* in *Arabidopsis thaliana* results in increased foliar sucrose/starch ratios and decreased foliar carbohydrate accumulation in plants after prolonged growth with CO_2_ enrichment [40]. Transformed tobacco plants overexpressing *Arabidopsis* SPS gene showed elevated transcript abundance and SPS enzyme activity, substantial pooling of soluble stem sucrose content, significantly increased stem height and greater stem diameters, longer fibers, and increased total dry biomass relative to control plants [41]. Vacuolar invertase (VINV) and cell wall invertase (CWINV, EC: 3.2.1.26) are invertases, cleaving sucrose to glucose and fructose. The transcripts and activities of *CWINV*, *CytINV*, and *VINV* at the fully mature stage were higher in Sweet Miriam, in agreement with the low sucrose content [42]. In our study, the expression of two *SPS* genes was downregulated and *CWINV1* expression was upregulated in abscised fruitlets in pecan, suggesting that sucrose synthesis was reduced.

Plant class I glucan endo-1,3-β-glucosidases (β-1,3-glucanase; 1,3-β-d-glucan glucanohydrolase, EC: 3.2.1.39) have been implicated in development and the defense against pathogen attack. β-glucosidases (EC: 3.2.1.21) are glycosyl hydrolases that hydrolyze the β-*O*-glycosidic bond at the anomeric carbon of a glucose moiety at the nonreducing end of a carbohydrate or glycoside molecule [43]. Endoglucanases (EC: 3.2.1.4) are associated with fruit ripening, growth of cultured cells, and leaf abscission. In our study, all 4 glucan *endo*-*1*,*3*-*β*-*glucosidase* genes, 6 out of 10 detected *β*-*glucosidase* genes, and a *endoglucanase* gene were downregulated in abscised fruitlets compared with persisting fruitlets, indicating that the glucose metabolism is blocked in abscised fruitlets in pecan.

Starch is the major storage carbohydrate in most plants, with many important functions. Granule-bound starch synthase (GBSS, EC: 2.4.1.242) is the glucosyl transferase specifically responsible for elongating amylose polymers and the only protein known to be required for its biosynthesis. Protein targeting of starch is required for localizing granule-bound starch synthase to starch granules and for normal amylose synthesis in Arabidopsis [44,45]. Two *GBSS1* genes and one *GBSS2* gene were downregulated in abscised fruitlets in pecan, indicating that starch biosynthesis was blocked. The 1,4-α-glucan branching enzyme (GBE, EC: 2.4.1.18) is known to cleave the α-1,4 glucosidic linkage of an existing glucan chain and transfer the cut end to the 6-position of a glucose residue within the cleaved chain or within another glucan chain, creating an α-1,6 glucosidic linkage, increasing the ratio of amylopectin to amylose [46]. Amylopectin has a slower recrystallization rate than amylose. GBE treatment could retard both short- and long-term retrogradation of starch in corn [47]. It is well known that a-amylases are important enzymes for starch degradation in plants [48]. In this study, two *GBE* genes were downregulated and two starch degradation alpha-amylase genes were upregulated in abscised fruitlets in pecan, showing that starch retrogradation and degradation are intensified. These results show that starch biosynthesis was blocked and starch retrogradation and degradation were intensified, resulting in reduced starch content in pecan.

Trehalose is an effective signaling molecule that has been shown to function in carbohydrate storage [49]. The trehalose biosynthetic pathway is used to transfer Glc from UDP-Glc to Glc-6-*P*, resulting in trehalose-6-*P* and UDP in plants. This initial step is catalyzed by TPS (EC: 2.4.1.15), and the dephosphorylation of trehalose-6-*P* occurs via TPP (EC: 3.1.3.12) producing trehalose in a second step [50]. *Arabidopsis* class I genes (AtTPS1–4) regulate starch storage, resistance to drought, and inflorescence architecture. Class II genes (AtTPS5–11) encode multifunctional enzymes with synthase and phosphatase activity [49], induced by sugar starvation [51]. Trehalose-6-phosphate, which is a sugar signal, induces a prompt reaction to nutritional stress during the early induction of abscission. The expression levels of a class II *TPS* gene were found to be high in the cortex of abscising fruitlets of apple and citrus [5]. Celton et al. reported that *TPP* and *TPS* genes showed increased expression in the pedicels of apple central fruitlets, which were persisting fruits, indicating that central pedicels may be supplied from a very early stage of development in apple [6]. Overexpression of *Escherichia coli TPS* (*OTS A*) in transgenic tobacco was shown to increase photosynthetic activity, but constitutive expression of *TPP* (*OTS B*) resulted in reduced photosynthesis [52]. However, the expression of *TPS* and *TPP* in was higher in abscised fruitlets than in persisting fruitlets in pecan in our study, indicating that the functions of trehalose may vary among plants, which needs further validation in the early stage of pecan fruitlet abscission.

Previous reports showed that galactose synthesis via alpha-galactosidase (EC: 3.2.1.22) results from the hydrolysis of raffinose to yield free galactose and sucrose [53]. α-*GAL* and β-*GAL* transcripts were higher in Santa Rosa fruits and leaves than in Sweet Miriam and correlated well with the higher Santa Rosa galactose content [42]. In our study, the expression of gene coding alpha-galactosidase was downregulated, but the expression of gene coding aldose 1-epimerase (EC: 5.1.3.3) was enhanced, indicating that galactose synthesis is reduced and degradation is enhanced in abscised fruitlets. UDP-Gal together with myo-inositol are used as substrates by GolS(EC: 4.1.123) for the synthesis of galactinol [54]. Three out of 4 genes coding *GolS* were downregulated in abscised pecan fruitlets, showing that galactinol synthesis was also blocked.

### 4.4. NAC Transcription Factors Participate in Fruit Development or Abscission Process

Multiple NAC family proteins were identified in abscised fruitlets in our study. The NAC proteins, which constitute one of the largest major transcription factor families, are well-known for their roles in several developmental programs [55]. NAC TFs have been shown to play important roles in various biological processes, as well as responses to abiotic stresses, and are important regulators in a wide range of developmental processes, such as the formation of lateral roots, the development of shoot apical meristem, floral morphogenesis, embryo development, grain nutrient remobilization, and cell wall biosynthesis [56]. In kiwifruit, 74 of 142 NACs were found to be persistently expressed in fruit during the whole developmental process, and crucial candidate *NAC* genes were shown to be involved in fruit growth and development [57]. Several NAC transcription factors were upregulated in the pedicels of central fruits, which were persisting fruits, in *Malus domestica*, which might contribute to increased vascular development in the central pedicel in the early developmental stages [6]. In our study, 24 NAC transcription factors were up- or downregulated in abscised fruitlets, indicating that these genes participate in the fruit development or abscission process.

## 5. Conclusions

The fruit development and drop dynamics of the pecan ‘Pawnee’ were studied in this paper, and the results show that the fruit drop rate was more than 57%, suggesting that fruit drop was very serious. In order to understand the mechanism of fruitlet drop, transcriptomic profiling of persisting and abscised fruitlets in early fruit development was conducted by RNA-seq. A total of 11,976 DEGs were identified, 3012 upregulated and 8964 downregulated, in the comparison of abscised vs. persisting fruitlets at 35 DAA. Our transcriptomic data suggest that a majority of the transcriptionally activated genes involved in hormone signaling were downregulated in the abscised fruitlets, except for ethylene and salicylic acid signaling. Unlike the downregulation of GA signaling specifically in fruits induced to abscise in apple [5], the GA content was increased in abscised fruitlets in pecan. Thus, the involvement of plant hormone signaling, especially GAs, at the early stage of fruit abscission should be studied in the future. Transcripts coding for enzymes involved in sucrose, glucose, trehalose, starch, galactose, and galactinol metabolism were reduced, suggesting that abscised pecan fruitlets stopped growing and developing for some time before dropping, indicating that their sugar supply was reduced or stopped. The transcriptome characterization described in this paper contributes to unravelling the molecular mechanisms and pathways involved in the physiological abscission of pecan fruits.

## Figures and Tables

**Table 1:** Sequencing and statistics of transcriptome data of two groups with reference genome (Cil.genome.fa).

Group Name	Persisting Fruitlets (P1)	Abscised Fruitlets (A1)
No. of clean bases (×10^8^)	68.24 ± 1.00	56.40 ± 0.69
No. of total reads (×10^6^)	45.30 ± 0.68	37.48 ± 0.47
No. of mapped reads (×10^6^)	42.57 ± 0.65	35.32 ± 0.40
Mapped percentage (%)	93.98 ± 0.0.3	94.25 ± 0.16
No. of unique mapped reads (×10^6^)	41.45 ± 0.62	34.39 ± 0.47
No. of sequenced reference genes	24,307 ± 73	21,723 ± 360
Percentage of sequenced reference genes (%)	78.23 ± 0.24	69.91 ± 1.16
No. of sequenced novel genes	1874.00 ± 14.73	1701.33 ± 22.68
Percentage of sequenced novel genes (%)	89.80 ± 0.71	81.52 ± 1.09
Sequenced total genes	26,181.00 ± 88.10	23,424.33 ± 382.98
Percentage of sequenced total genes (%)	78.96 ± 0.27	70.64 ± 1.15

**Table 2:** KEGG pathways mapping enriched differential progress (*p* < 0.01).

Pathway	DEGs	*p*-Value	q-Value	Pathway ID
Biosynthesis of secondary metabolites	306	8.64 × 10^−8^	1.11 × 10^−5^	ko01110
Metabolic pathways	492	1.50 × 10^−6^	9.69 × 10^−5^	ko01100
Plant–pathogen interaction	120	9.58 × 10^−5^	0.004	ko04626
Monoterpenoid biosynthesis	11	2.94 × 10^−4^	0.009	ko00902
Starch and sucrose metabolism	41	6.91 × 10^−4^	0.018	ko00500
Valine, leucine, and isoleucine degradation	19	1.99 × 10^−3^	0.043	ko00280
Carotenoid biosynthesis	16	2.40 × 10^−3^	0.044	ko00906
DNA replication	18	3.64 × 10^−3^	0.059	ko03030
Galactose metabolism	22	5.96 × 10^−3^	0.083	ko00052
Plant hormone signal transduction	67	6.40 × 10^−3^	0.083	ko04075

**Table 3:** Identified differentially expressed proteins involved in plant hormone signal transduction (ko04075).

Gene ID	Description	Symbol	Name	log2(A/P)	*p*-Value
Abscisic acid					
CIL1308S0009	Abscisic acid receptor PYL9	PYR/PYL	PYL9	−1.938	4.18 × 10^−15^
CIL1506S0033	Protein phosphatase 2C 53	PP2C	HAB1	−2.868	6.3 × 10^−9^
CIL1317S0086	Protein phosphatase 2C 16	PP2C	HAB1	−1.024	0.029075
CIL1562S0028	Protein phosphatase 2C 75	PP2C	AHG1	−2.910	2.89 × 10^−8^
CIL0302S0016	Serine/threonine-protein kinase SAPK7	SnRK2	SRK2H	−2.478	6.01 × 10^−8^
CIL0409S0006	Serine/threonine-protein kinase SAPK2	SnRK2	SAPK2	−7.128	2.1 × 10^−9^
CIL0045S0005	ABSCISIC ACID-INSENSITIVE 5-like protein 5	ABF	DPBF3	2.287	1.66 × 10^−59^
CIL1371S0045	ABSCISIC ACID-INSENSITIVE 5-like protein 6	ABF	ABF2	−2.671	1.13 × 10^−6^
CIL1387S0048	ABSCISIC ACID-INSENSITIVE 5-like protein 2	ABF	DPBF3	−1.484	0.000172
Auxin					
CIL1565S0004	Auxin transporter-like protein 3	AUX1	LAX3	1.772	1.09 × 10^−7^
CIL1464S0004	TRANSPORT INHIBITOR RESPONSE protein	TIR	TIR1	−2.584	3.62 × 10^−6^
CIL0202S0015	Auxin-responsive protein IAA16-like	AUX/IAA	IAA16	−16.109	3.02 × 10^−23^
CIL0203S0027	Auxin-responsive protein IAA9-like	AUX/IAA	IAA9	−3.587	1.04 × 10^−39^
CIL0344S0014	Auxin-responsive protein IAA18-like	AUX/IAA	IAA26	−2.671	2.16 × 10^−11^
CIL0732S0001	Auxin-responsive protein IAA9-like	AUX/IAA	IAA9	−10.617	0.000112
CIL1268S0077	Auxin-responsive protein IAA27-like	AUX/IAA	IAA8	−3.986	6.35 × 10^−5^
CIL1294S0084	Auxin-responsive protein IAA20-like	AUX/IAA	IAA20	−9.765	0.001964
CIL1320S0049	Auxin-responsive protein IAA27-like	AUX/IAA	IAA27	−4.217	2.06 × 10^−11^
CIL1358S0014	Auxin-responsive protein IAA29-like	AUX/IAA	IAA11	−10.951	8.53 × 10^−7^
CIL1490S0013	Auxin response factor 18	ARF	ARF9	−8.323	1.83 × 10^−54^
CIL1564S0002	Auxin response factor 9	ARF	ARF9	−3.530	1.08 × 10^−38^
CIL1354S0026	Auxin response factor 19	ARF	ARF7	−2.515	3.06 × 10^−13^
CIL1313S0055	Probable indole-3-acetic acid-amido synthetase GH3.6	GH3	GH3.6	2.440	5.86 × 10^−18^
CIL1405S0075	Indole-3-acetic acid-amido synthetase GH3.6	GH3	GH3.6	−4.055	6.98 × 10^−7^
CIL1456S0019	Probable indole-3-acetic acid-amido synthetase GH3.1	GH3	GH3.1	−2.721	1.89 × 10^−6^
CIL1295S0016	Auxin-responsive protein SAUR36	SAUR	SAUR36	3.508	3.6 × 10^−26^
CIL0367S0005	Auxin-induced protein X15	SAUR	SAUR50	−3.078	0.003923
MSTRG.7503	Auxin-responsive protein SAUR32	SAUR	SAUR32	6.343	4.96 × 10^−90^
CIL1294S0056	Indole-3-acetic acid-induced protein ARG7	SAUR	SAUR36	10.129	0.000393
Cytokinin					
CIL1595S0016	Histidine kinase 2	CRE1	AHK2	−4.205	3.31 × 10^−19^
CIL1384S0015	Histidine kinase 2	CRE1	AHK2	−1.934	0.004026
CIL0037S0021	Histidine-containing phosphotransfer protein 1	AHP	AHP1	−12.078	3.11 × 10^−9^
CIL1268S0039	Histidine-containing phosphotransfer protein 4	AHP	PHP5	−6.299	2.73 × 10^−5^
CIL1268S0040	Histidine-containing phosphotransfer protein 4	AHP	AHP4	10.326	1.02 × 10^−6^
CIL1369S0025	Histidine-containing phosphotransfer protein 1	AHP	AHP1	−1.748	3.06 × 10^−9^
MSTRG.14062	Histidine-containing phosphotransfer protein 1	AHP	AHP1	1.101	0.001041
CIL1575S0022	Two-component response regulator ARR12	B-ARR	RR23	−2.035	5.52 × 10^−13^
CIL0004S0012	Two-component response regulator ARR8	A-ARR	ARR8	−11.423	0.000187
CIL0338S0017	Two-component response regulator ARR9	A-ARR	ARR9	−1.943	0.030964
CIL1308S0026	Two-component response regulator ARR5	A-ARR	ARR4	−4.320	2.19 × 10^−12^
CIL1596S0045	Two-component response regulator ARR5	A-ARR	ARR15	−5.520	3.19 × 10^−12^
	Ethylene				
CIL1354S0046	Ethylene-responsive transcription factor 1B	EBF1/2	ERF1B	2.436	0.000283
CIL1358S0043	EIN3-binding F-box protein 1	EBF1/2	EBF2	2.280	7.01 × 10^−36^
CIL1493S0026	Ethylene receptor 2	ETR	ETR2	4.072	1.15 × 10^−73^
Gibberellin					
CIL1324S0067	DELLA protein SLN1	DELLA	GAI1	−3.061	1.6 × 10^−16^
CIL1294S0080	Transcription factor PIF3	TF	PIL15	−5.458	8.24 × 10^−14^
CIL1495S0010	Transcription factor PIF1	TF	PIF1	2.298	5.46 × 10^−8^
Brassinosteroid					
CIL1492S0038	BRASSINOSTEROID INSENSITIVE 1-associated receptor kinase 1	BAK1	BAK1	1.609	1.12 × 10^−22^
CIL1312S0044	Brassinosteroid LRR receptor kinase	BRI1	CURL3	−8.773	1.46 × 10^−58^
MSTRG.23225	Serine/threonine-protein kinase	BSK	BSK1	−1.699	0.003421
CIL1383S0002	BES1/BZR1 homolog protein 2	BZR1/2	BEH2	−1.441	0.013346
CIL0182S0015	HMA domain-containing protein	BZR1/2	–	5.157	0.000157
CIL1577S0019	Xyloglucan endotransglucosylase/hydrolase protein 22	TCH4	XTH22	−3.772	2.16 × 10^−5^
CIL1371S0059	Cyclin-D3-1	CYCD3	CYCD3-3	−11.257	4 × 10^−9^
JA					
CIL1295S0055	Protein TIFY 10A	JAZ	TIFY10B	2.236	3.57 × 10^−13^
CIL1338S0009	Protein TIFY 6B	JAZ	TIFY6B	−4.081	4.52 × 10^−18^
CIL1390S0024	Protein TIFY 6B	JAZ	TIFY6B	−5.604	1.59 × 10^−14^
CIL1565S0018	Protein TIFY 6B	JAZ	TIFY6B	−3.637	1.25 × 10^−12^
MSTRG.9204	Protein TIFY 10A	JAZ	–	−3.812	0.016461
CIL1399S0034	Transcription factor MYC2	MYC2	BHLH14	−3.273	9.07 × 10^−10^
MSTRG.9232	Transcription factor MYC4	MYC2	MYC4	6.314	2 × 10^−22^
SA					
CIL1326S0021	Transcription factor TGA7-like	TGA	TGA7	1.407	1.05 × 10^−15^
CIL0037S0002	Pathogenesis-related protein 1	PR-1	PRB1	8.827	1.24 × 10^−60^
CIL0037S0003	Pathogenesis-related protein 1	PR-1	PRB1	3.015	1.31 × 10^−16^
CIL0037S0006	Pathogenesis-related protein 1	PR-1	At2g14610	8.831	1.4 × 10^−139^
CIL0037S0007	Basic form of pathogenesis-related protein 1	PR-1	PRMS	10.196	6.07 × 10^−5^
CIL0232S0001	Pathogenesis-related protein 1	PR-1	PRB1	−4.242	9.5 × 10^−24^

**Table 4:** Response of transcription factors in comparison of A1 vs. P1.

Category	Total	Upregulated	Downregulated
MYB	40	10	30
NAC	24	13	11
bHLH	24	4	20
WRKY	24	17	7
bZIP	4	1	3
C2	2	0	2
C2H2	1	0	1
MADS	3	0	3
ERF	25	11	14
AUX/IAA	25	4	21
zinc finger	69	15	54
B3	4	1	4

**Table 5:** Starch and sucrose metabolism and galactose metabolism.

Gene ID	Description	EC No.	Symbol	log2(fc)	*p*-Value
CIL1395S0001	Alpha-glucosidase	3.2.1.20	–	1.594	0.012
MSTRG.2545	Alpha-glucosidase	3.2.1.20	Os06g0675700	1.429	2.71 × 10^−8^
CIL1595S0049	Glucose-1-phosphate adenylyltransferase	2.7.7.27	glgC	−2.746	2.81 × 10^−16^
CIL0203S0004	Glucose-1-phosphate adenylyltransferase	2.7.7.27	glgC	−3.281	1.24 × 10^−10^
MSTRG.12720	Alpha-amylase-like isoform X1	3.2.1.1	AMY1.1	3.719	1.74 × 10^−76^
MSTRG.2528	Alpha-amylase-like isoform X1	3.2.1.1	AMY1.1	1.358	6.01 × 10^−13^
CIL1383S0034	Glucan endo-1,3-beta-glucosidase 1	3.2.1.39	At1g11820	−3.735	1.49 × 10^−32^
CIL1482S0014	Glucan endo-1,3-beta-glucosidase 4-like	3.2.1.39	At3g13560	−3.409	2.95 × 10^−15^
CIL1332S0070	Glucan endo-1,3-beta-glucosidase 5-like	3.2.1.39	At4g31140	−1.632	4.40 × 10^−4^
CIL1347S0008	Glucan endo-1,3-beta-glucosidase 6	3.2.1.39	At5g58090	−6.006	5.41 × 10^−32^
CIL1359S0017	Hexokinase-3-like [Juglans regia]	2.7.1.1	At1g50460	−1.211	3.43 × 10^−3^
MSTRG.14644	Hexokinase-3-like isoform X3	2.7.1.1	At1g50460	−2.125	2.03 × 10^−7^
CIL1459S0007	Hexokinase-3-like isoform X2	2.7.1.1	At1g50460	−2.411	5.93 × 10^−7^
CIL0282S0003	Hexokinase-1-like isoform X2	2.7.1.1	HXK1	1.958	1.53 × 10^−12^
CIL1518S0008	Hexokinase-2	2.7.1.1	HXK2	−2.598	0.005
CIL1568S0010	Probable fructokinase-7	2.7.1.4	At5g51830	2.623	2.65 × 10^−36^
CIL0508S0004	Beta-glucosidase 12-like isoform X3	3.2.1.21	BGLU12	−3.745	0.003
CIL1537S0001	Beta-glucosidase 12-like	3.2.1.21	BGLU12	−8.471	5.16 × 10^−51^
CIL0493S0002	Beta-glucosidase 12-like	3.2.1.21	BGLU13	11.919	2.12 × 10^−11^
MSTRG.6474	Beta-glucosidase 12-like	3.2.1.21	BGLU13	10.737	1.03 × 10^−7^
CIL0508S0002	Beta-glucosidase 13-like isoform X2	3.2.1.21	BGLU13	−2.922	1.26 × 10^−4^
MSTRG.7161	Beta-glucosidase 12-like	3.2.1.21	BGLU24	−9.010	1.78 × 10^−54^
CIL1405S0071	Beta glucosidase 41 isoform 2	3.2.1.21	BGLU25	−3.444	1.12 × 10^−11^
CIL0391S0004	Beta-glucosidase 42 isoform X1	3.2.1.21	BGLU42	−4.571	2.02 × 10^−52^
CIL1320S0039	Beta-glucosidase 47-like isoform X1	3.2.1.21	BGLU47	4.020	9.97 × 10^−18^
CIL1407S0038	Endoglucanase 8-like	3.2.1.4	CEL1	−6.822	8.06 × 10^−27^
MSTRG.23283	Beta-glucosidase	3.2.1.21	RE1	2.092	1.75 × 10^−3^
CIL1317S0076	Beta-fructofuranosidase	3.2.1.26	CWINV1	4.017	8.53 × 10^−14^
CIL1506S0011	Beta-fructofuranosidase	3.2.1.26	CWINV3	−5.878	1.01 × 10^−4^
CIL1264S0043	Acid beta-fructofuranosidase-like	3.2.1.26	INV*DC4	−3.426	1.78 × 10^−25^
CIL0525S0001	Nudix hydrolase 14, chloroplastic	3.6.1.21	NUDT14	−1.662	8.32 × 10^−4^
CIL1568S0006	Phosphoglucomutase, chloroplastic	5.4.2.2	PGMP	−1.205	0.011
CIL0360S0002	Sucrose-phosphate synthase 1	2.4.1.14	SPS1	−1.765	8.57 × 10^−3^
CIL1271S0008	Sucrose-phosphate synthase 1	2.4.1.14	SPS1	−7.197	1.16 × 10^−29^
CIL1417S0045	1,4-alpha-glucan-branching enzyme 3	2.4.1.18	GBE3, glgB, SBE3	−4.466	5.87 × 10^−16^
CIL1531S0004	1,4-alpha-glucan-branching enzyme 1	2.4.1.18	GBE3, glgB, SBEI	−2.321	4.31 × 10^−18^
CIL0218S0017	Granule-bound starch synthase 1	2.4.1.242	WAXY, GBSS1	−3.748	8.21 × 10^−14^
CIL0389S0009	Granule-bound starch synthase 1	2.4.1.242	WAXY, GBSS1	−12.135	6.74 × 10^−12^
CIL0176S0049	Granule-bound starch synthase 2	2.4.1.21	SS2	−5.083	1.69 × 10^−20^
CIL1531S0021	Trehalose-phosphate phosphatase	3.1.3.12	TPP	1.896	3.70 × 10^−5^
CIL1310S0034	Trehalose 6-phosphate synthase	2.4.1.15	TPS	1.535	1.66 × 10^−12^
CIL0021S0018	Alpha-galactosidase 1-like	3.2.1.22	AGAL1	−2.014	3.88 × 10^−12^
CIL1429S0021	Aldose 1-epimerase	5.1.3.3	Galm	1.077	4.95 × 10^−48^
CIL0309S0003	Inositol 3-alpha-galactosyltransferase 1	2.4.1.123	GOLS1	1.063	1.44 × 10^−11^
MSTRG.21476	Inositol 3-alpha-galactosyltransferase 1	2.4.1.123	GOLS1	−13.936	3.70 × 10^−15^
MSTRG.20754	Inositol 3-alpha-galactosyltransferase 2	2.4.1.123	GOLS2	−2.722	0.013
MSTRG.20755	Inositol 3-alpha-galactosyltransferase 2	2.4.1.123	GOLS2	−2.837	0.021
CIL0344S0021	6-phosphofructokinase 1	2.7.1.1	PFK3	−4.090	2.05 × 10^−8^
CIL1568S0006	Phosphoglucomutase	5.4.2.2	PGMP	−1.205	0.011
CIL1358S0005	Raffinose synthase	2.4.1.82	RFS6	3.211	2.01 × 10^−33^
CIL0272S0009	UDP-glucose 4-epimerase GEPI48-like	5.1.3.2	UGE5	1.349	1.70 × 10^−11^
CIL1564S0017	UDP-glucose 4-epimerase GEPI48	5.1.3.2	UGE5	3.159	8.72 × 10^−43^
CIL1297S0013	UDP-sugar pyrophosphorylase	2.7.7.64	USP	−3.752	4.18 × 10^−8^

## Data Availability

The data presented in this study are openly available in NCBI SRA (http://www.ncbi.nlm.nih.gov/sra), reference number [PRJNA784729].

## Supplementary Materials

The following are available online at https://www.mdpi.com/article/10.3390/cimb44010013/s1.
