# Supplementary material for: Comparative Analysis of the Transcriptomes of Persisting and Abscised Fruitlets: Insights into Plant Hormone and Carbohydrate Metabolism Regulated Self-Thinning of Pecan Fruitlets during the Early Stage
Source: Curr Issues Mol Biol. 2021 Dec 30;44(1):176–93. doi: 10.3390/cimb44010013 (PMC8929008; doi:10.3390/cimb44010013)
Supplement: Supplementary file 1 [file cimb-44-00013-s001.zip › cimb-1467237-supplementary materials.pdf]

Supplementary Materials

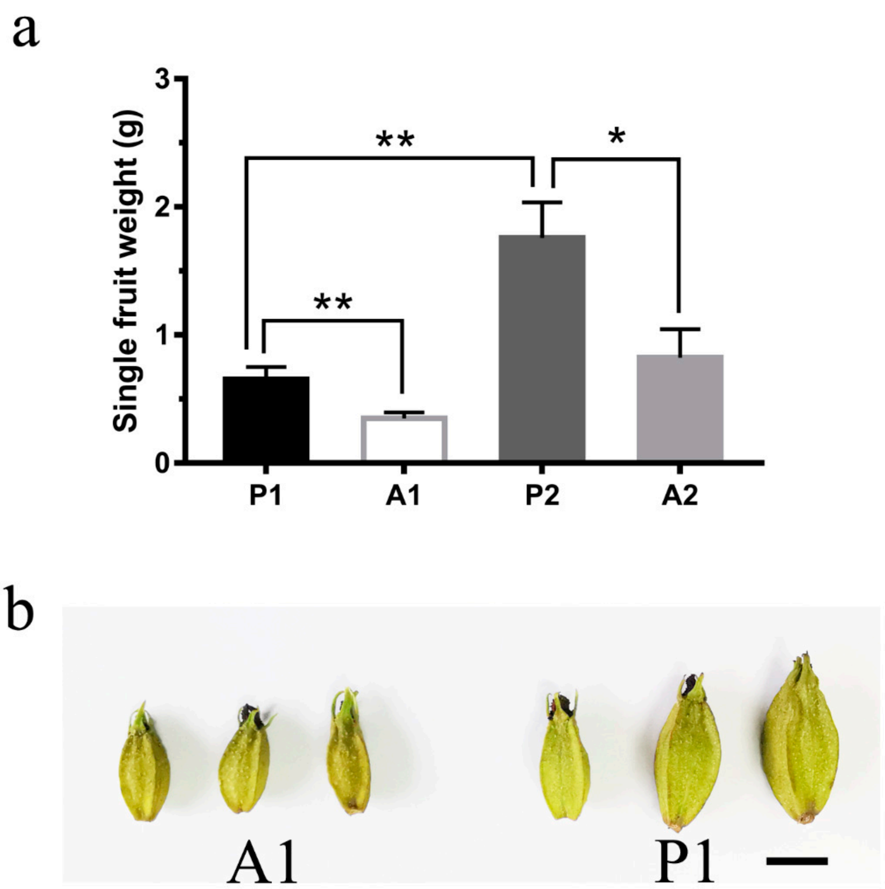

Supplementary Figure S1. Single fruit weight (**a**) and phenotype (**b**) of P1 and A1 in pecan. Statistically significant differences were calculated with GraphPad Prism 7.00. Data represent average ± SD of three biological repeats with three measurements. * Significant differences at $p < 0.05$; ** significant differences at $p < 0.01$.

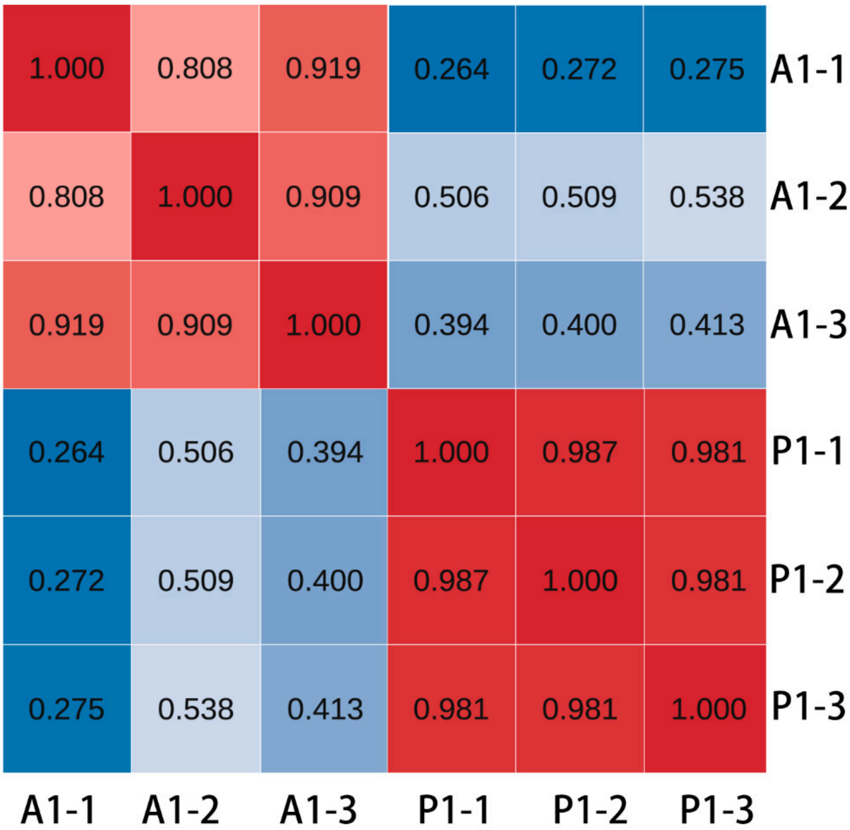

Supplementary Figure S2. The correlation coefficient in the repeat group of A1 and P1.
